# Diversity of miniaturized frogs of the genus *Adelophryne* (Anura: Eleutherodactylidae): A new species from the Atlantic Forest of northeast Brazil

**DOI:** 10.1371/journal.pone.0201781

**Published:** 2018-09-19

**Authors:** Ricardo Lourenço-de-Moraes, Iuri R. Dias, Caio V. Mira-Mendes, Renan M. de Oliveira, Adriane Barth, Danilo S. Ruas, Miguel Vences, Mirco Solé, Rogério P. Bastos

**Affiliations:** 1 Departamento de Ecologia, Instituto de Ciências Biológicas, Laboratório de Herpetologia e Comportamento Animal, Campus Samambaia, Universidade Federal de Goiás, Goiânia, Goiás, Brazil; 2 Programa de Pós-Graduação em Zoologia, Universidade Estadual de Santa Cruz, Ilhéus, Bahia, Brazil; 3 Departamento de Ciências Biológicas, Universidade Estadual de Santa Cruz, Ilhéus, Bahia, Brazil; 4 Programa de Pós-Graduação em Sistemas Aquáticos Tropicais, Universidade Estadual de Santa Cruz, Ilhéus, Bahia, Brazil; 5 Programa de Pós-Graduação em Ecologia e Conservação da Biodiversidade, Universidade Estadual de Santa Cruz, Ilhéus, Bahia, Brazil; 6 Departamento de Vertebrados, Museu Nacional, Universidade Federal do Rio de Janeiro, Rio de Janeiro, Brazil; 7 Departamento de Ensino, Instituto Federal de Educação Ciência e Tecnologia do Estado de Mato Grosso, Rondonópolis, Mato Grosso, Brazil; 8 Departamento de Ciências Naturais, Universidade Estadual do Sudoeste da Bahia, Itapetinga, Bahia, Brazil; 9 Zoological Institute, Technische Universität Braunschweig, Braunschweig, Germany; Universitat Trier, GERMANY

## Abstract

The number of species of frogs in the South American genus *Adelophryne* has increased in recent years, and it has become apparent that this group contains a substantial amount of undescribed diversity. Currently the genus contains nine described species and five candidate species. Here we describe the tenth species of the genus *Adelophryne* from the municipality of Igrapiúna, southern Bahia state, Brazil. The new species is characterized by its small body size, indistinct tympanum, and two phalanges in the finger IV. The species of the genus are distributed in three groups, Northern Amazonia Clade, Northern Atlantic Forest Clade and Southern Atlantic Forest Clade. The new species is phylogenetically related to species of the Northern Atlantic Forest Clade of *Adelophryne* and restricted to forested habitat, as typical for other *Adelophryne*. The species is restricted to the pristine forests in the type locality, and we consider its conservation status as Near Threatened. New morphological and molecular data of other *Adelophryne* species are presented, extending the distribution of *Adelophryne* sp. 2, *Adelophryne* sp. 4, *Adelophryne mucronata* and *Adelophryne glandulata*. However, a more comprehensive revision of the diversity and phylogenetic position of most *Adelophryne* species is needed, and the evolutionary relationships of *A*. *meridionalis* and *A*. *pachydactyla* remain unknown.

## Introduction

The genus *Adelophryne* Hoogmoed & Lescure, [1] contains nine poorly known species of diminutive frogs inhabiting leaf litter of forests, with discontinuous distribution in eastern Brazil, the Guiana Shield, and the upper Amazon Basin [2,3]. Ten years after the description of the genus, five species were known: *Adelophryne adiastola* Hoogmoed & Lescure, [1]; *Adelophryne baturitensis* Hoogmoed, Borges & Cascon, [4]; *Adelophryne gutturosa* Hoogmoed & Lescure, [1]; *Adelophryne maranguapensis* Hoogmoed, Borges & Cascon, [4]; and *Adelophryne pachydactyla* Hoogmoed, Borges & Cascon, [4]. However, since 2008, four new species have been described: *Adelophryne patamona* MacCulloch, Lathrop, Kok, Minter, Khan & Barrio-Amorós, [5]; *Adelophryne mucronata* Lourenço-de-Moraes, Solé & Toledo, [6]; *Adelophryne meridionalis* Santana, Fonseca, Neves & Carvalho, [7] and *Adelophryne glandulata* Lourenço-de-Moraes, Ferreira, Fouquet & Bastos, [8]. A molecular phylogeny of the *Adelophryne* species, inferred by Fouquet et al. [9] confirmed the monophyly of the genus and revealed the existence of seven additional candidate species of which five remain undescribed. According to Fouquet et al. [9] the genus is represented by three deeply divergent and well-sustained clades that are geographically circumscribed to the Northern Amazonia Clade (NAMC), Northern Atlantic Forest Clade (NAFC) (from Ceará to northern Bahia) and Southern Atlantic Forest Clade (SAFC) (from southern Bahia, Minas Gerais and Espírito Santo).

In this paper, we describe a new species of the genus *Adelophryne* from the Atlantic Rain Forest in southern Bahia state, northeast Brazil and provide information on its natural history, molecular phylogenetic relationships, and conservation status. Additionally, based on the molecular data we update the geographic distribution of *Adelophryne* sp. 2, *A*. sp. 4, *A*. *mucronata* and *A*. *glandulata*, and provide new phylogenetic and taxonomic information for *A*. *pachydactyla*, and *A*. sp. 5 (*sensu* Fouquet et al. [9]).

## Materials and methods

### Ethics statement

This study was conducted with appropriate permissions and guidelines from the responsible authority—licence (13708) issued by “Instituto Chico Mendes de Conservação da Biodiversidade” (ICMBio) that also evaluates protocols for our collection and research. Specimens were collected by hand, euthanized by overdose of an anesthetic solution (5% lidocaine), fixed in 10% formalin, and preserved in 70% ethyl alcohol. Muscle samples were taken from thighs and stored in absolute ethanol for subsequent DNA extraction and sequencing. Collected specimens were not recognized as belonging to threatened species and they are not listed in the Red List of Threatened Species of the International Union for Conservation of Nature (IUCN), or in the appendices of the Convention on International Trade in Endangered Species of Wild Fauna and Flora (CITES). This research was approved by the ethics committee on the use of animals (CEUA-UESC 002/12).

### Study sites

The new species was found in forest fragments at the Reserva Ecológica Michelin (REM—13°49’15”S, 39°11’52”W, 95 m above sea level), located in the south of Bahia, municipality of Igrapiúna. According to Veloso et al. [10] the region is characterized as Tropical Lowland Rain Forest. The REM comprises a total area of 3096 ha, of which 1800 ha are made up by three tropical rainforest fragments. Most of the forest is secondary, at different stages of succession, and in part intensively logged, with small patches of intact forest areas on hillsides and hilltops with range of 40–586 m above sea level [11]. According to Köppen-Geiger’s climate classification the region is of type AF [12].

All specimens of the new species were collected at REM. Voucher specimens were deposited in the Museu de Zoologia da Universidade Estadual de Santa Cruz, Ilhéus (MZUESC), Coleção Zoológica da Universidade Federal de Goiás (ZUFG), and Museu de Biologia Mello Leitão (MBML).

### Protocol of species delimitation

We used the terminology for morphological characters, and the description format of Hoogmoed et al. [4], Lourenço-de-Moraes et al. [6,8], Cei [13], Heyer et al; [14], and Kok & Kalamandeen [15]; more details are provided below. To prepare cleared and stained individuals we used the protocol by Taylor & Van Dyke [16]. Sex was determined based on gonads or by the presence of eggs in females or vocal sac folds in males. Drawings of holotype (by Raoni Rebouças) were done using a digital board Wacom Cintiq 21UX and Autodesk Sketchbook ver. 4.1.6 software.

### Morphological analysis and specimens

Measurements were taken under a stereomicroscope with digital calipers. The measurements follow Lourenço-de-Moraes et al. [6,8]: snout–vent length (SVL); head length (HL); head width (HW); eye diameter (ED); upper eyelid width (UEW); interorbital distance (IOD); internarial distance (IND); eye-nostril distance (END); nostril to tip of snout distance (NSD); eye to tip of snout distance (ETSD); foot length (FL); thigh length (THL); and tibia length (TL). The description of snout shape in lateral view follows Cei [13] and terminology of dorsal view follows Heyer et al. [14], terminology of the tympanum, fingers, toes and pads follows Hoogmoed et al. [4] and Lourenço-de-Moraes et al. [6] for terminal tips; terminology of skin texture follows Kok & Kalamandeen [15]. Skeletal characters were determined from two cleared and stained [16] individuals (ZUFG 10695–10696). We dissected five specimens for stomach content analysis (ZUFG 10695–10697, MZUESC 17506, MBML10498) with the aid of a stereomicroscope.

Comparisons between species were made using original species descriptions [1,4–8] and direct examination of specimens from the following collections: Célio F.B. Haddad Collection, Universidade Estadual Paulista—CFBH, División de Herpetología, Museo Ecuatoriano de Ciencias Naturales, Quito, Ecuador—DHMECN, Museu Nacional do Rio de Janeiro—MNRJ, Museu de Zoologia João Moojen, Universidade Federal de Viçosa—MZUFV, Museu de Zoologia da Universidade Estadual de Campinas “Adão José Cardoso”—ZUEC, Museu de Zoologia da Universidade Estadual de Santa Cruz—MZUESC, Museu de Biologia Mello Leitão—MBML, Coleção Zoológica da Universidade Federal de Goiás—ZUFG, Coleção herpetológica da Universidade Federal de Minas Gerais -UFMG and Núcleo Regional de Ofiologia, Laboratório de Herpetologia da Universidade Federal do Ceará- NUROF-UFC (see S1 Appendix for details).

### Molecular procedures

We extracted total genomic DNA using a standard salt extraction protocol [17] from leg muscle of two individuals of *Adelophryne* sp. nov, two samples of *Adelophryne mucronata* and a tissue from a third unidentified species from the Reserva Ecológica Michelin (REM), Igrapiúna, Bahia. Furthermore, we included thirteen samples from other species collected from seven localities in southern Bahia, and from a paratype of *A*. *glandulata* (MZUESC 12180) from Santa Tereza, Espírito Santo. Sequences from partial mitochondrial 16S rDNA were amplified using published primer sets AC16SAR (F): CGCCTGTTTATCAAAAACAT and 16Sbr-H (R): CCGGTCTGAACTCAGATCACGT [18] for *Adelophryne* sp. nov. (MZUESC 19139 and ZUFG 10696) and two samples of *A*. *mucronata* (MZUESC 19140 and MZUESC 19141) from REM and all others with 16SC (F): GTRGGCCTAAAAGCAGCCAC [19] and 16SBr-H (R): CCGGTCTGAACTCAGATCACGT [18], same primers used by Fouquet et al. [9].

PCR Reactions were performed in a final volume of 11.5 μl using the following concentration: 0.3 μl of each primer, 0.25 μl of dNTP, 2.5 μl PCR buffer, and 0.1 μl of GoTaq DNA polymerase (Promega, Mannheim, Germany) and 1 μl of DNA. Amplification conditions consisted of a pre-denaturation step of 3 min at 92 °C, followed by 38 cycles of a denaturation step of 3 min at 92 °C, annealing at 48 °C for 50 sec and extension at 72 °C of 3 min. PCR products were purified with enzymatic process: 0.15 units of Shrimp Alkaline Phosphatase (SAP) and 1 unit of Exonuclease I (New England Biolabs, Frankfurt am Main, Germany) incubated for 15 min at 37°C followed by 15 min at 80°C. Purified PCR products were sequenced in an automated DNA sequencer (Applied Biosystems ABI 3130XL). Sequences were checked and edited using CodonCode Aligner 3.7.1(CodonCode Corporation, Dedham, MA, USA).

### Phylogenetic analyses

We generated static alignments in MAFFT [20] with the L-INS-i strategy. New sequences were submitted to Genbank (accession numbers: MH304333–MH304352 –S2 Appendix). Maximum likelihood (ML) algorithm and Bayesian inference (BI) were used for phylogenetic analysis. The ML analysis was performed in RAxML-HPC BlackBox 8.2.10 [21] under the GTRAC model and with 1000 bootstrap replicates. We performed BI analyses using MrBayes 3.2.3[22]. The models of molecular evolution were determined using Partition Finder 1.1.1 [23]. The GTR + I + G substitution model was selected as the optimal nucleotide substitution model for the 16S rRNA data set. Bayesian analyses included two independent runs, each with four chains and sampling every 1000 generations for 60 million generations. We examined trace plots and effective sample size (ESS) in Tracer v1.6 to determine MCMC mixing and convergence. We removed trees from the first 20% of the samples as burn-in. A consensus of the post-burning trees was visualized in Fig Tree v1.4.2. Both ML and BI analyses were performed at CIPRES Science Gateway [24]. For both phylogenetic analyses we used samples of species of the families Ranidae, Brachycephalidae, Craugastoridae and Eleutherodactylidae as outgroups (see S2 Appendix). Uncorrected p-distances were calculated for species of the genus *Adelophryne* from the DNA matrix using MEGA 5.1 [25].

All sequences of the mitochondrial 16S rRNA gene available in GenBank (http://www.ncbi.nlm.nih.gov/genbank/) for *Phyzelaphryne* and *Adelophryne* species used by Fouquet et al. [9] were also included in our alignment, except for one candidate species (*Adelophryne* sp. 3) from that study for which no 16S sequences were available.

### Nomenclatural acts

The electronic edition of this article conforms to the requirements of the amended International Code of Zoological Nomenclature, and hence the new names contained herein are available under that Code from the electronic edition of this article. This published work and the nomenclatural acts it contains have been registered in ZooBank, the online registration system for the ICZN. The ZooBank LSIDs (Life Science Identifiers) can be resolved and the associated information viewed through any standard web browser by appending the LSID to the prefix “http://zoobank.org/". The LSID for this publication is: urn:lsid:zoobank.org:act:C5966E51-D5DA-4B8E-BF14-73DBAF6C0163. The electronic edition of this work was published in a journal with an ISSN, and has been archived and is available from the following digital repositories: PubMed Central and LOCKSS.

### Status conservation and map of species distribution

Based on the natural history and distribution of the new species, we evaluated its possible conservation status using the criteria of the International Union for the Conservation of Nature (http://www.iucnredlist.org/). We hope this initial assessment will inform the IUCN to decide on the status of this species on the red list.

We used ArcGIS 10.1 software [26] for the elaboration of distribution maps for *Adelophryne* species in the Atlantic Forest, Brazil.

## Results

### Adelophryne michelin sp. nov.

*Adelophryne* sp. (Mira-Mendes et al. [27])

urn:lsid:zoobank.org:pub:774C6B9F-6453-47D7-9E55-2CC19886FEEE

#### Etymology

The name “*michelin”* honors the Reserva Ecológica Michelin that has been supporting our researches for more than 10 years in the municipality of Igrapiúna, Bahia. The name is used as an invariable noun in apposition to the generic name.

#### Common name

Michelin Flea Frog or rãzinha-pulga-da-Michelin (in Portuguese).

#### Holotype

**Fig 1:**
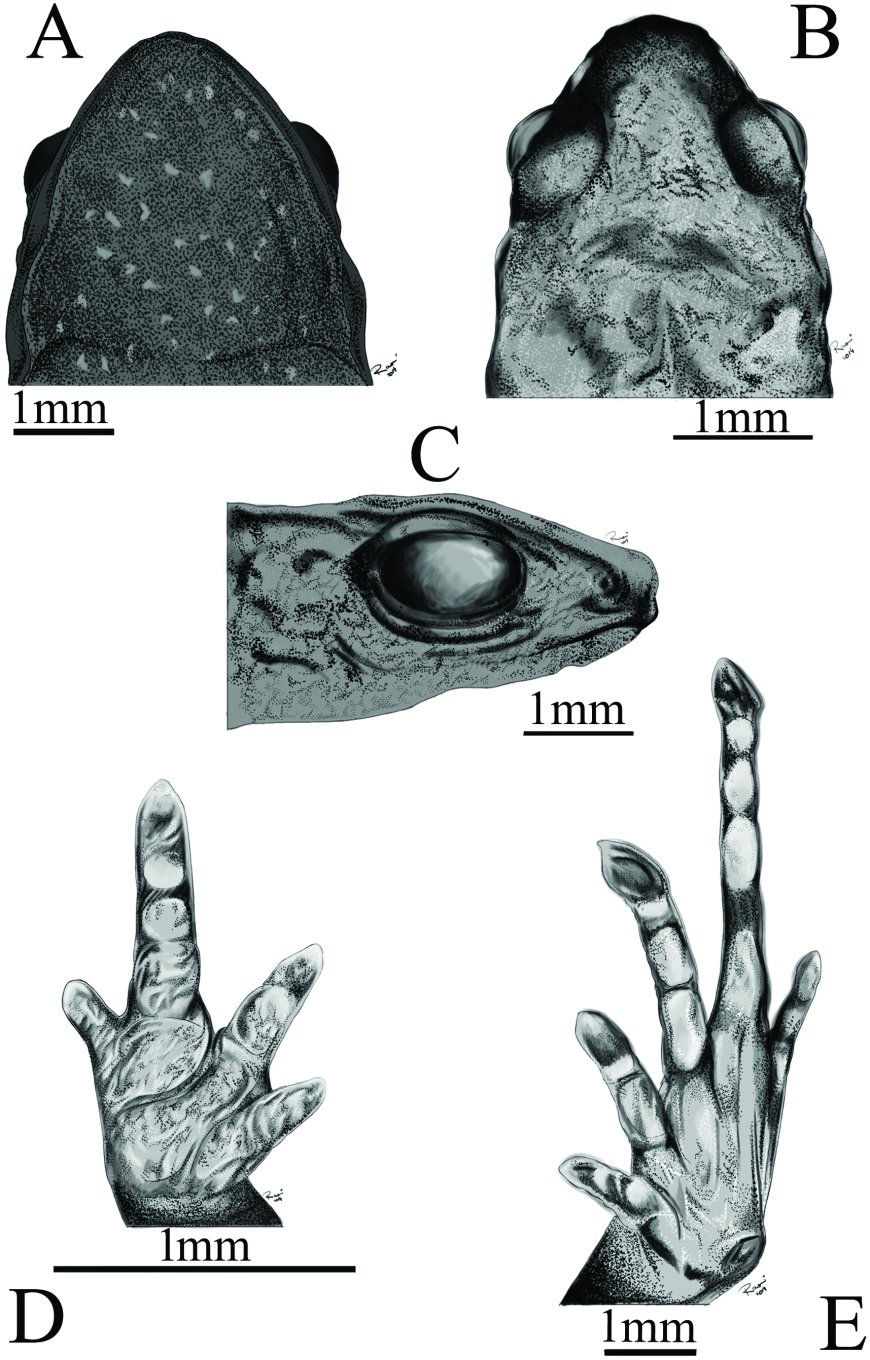
Adult holotype of *Adelophryne michelin* sp. nov. (MZUESC 17509, SVL 10.5 mm). Ventral (A), dorsal (B), and lateral (C) views of head, ventral views of hand (D) and foot (E).

**Fig 2:**
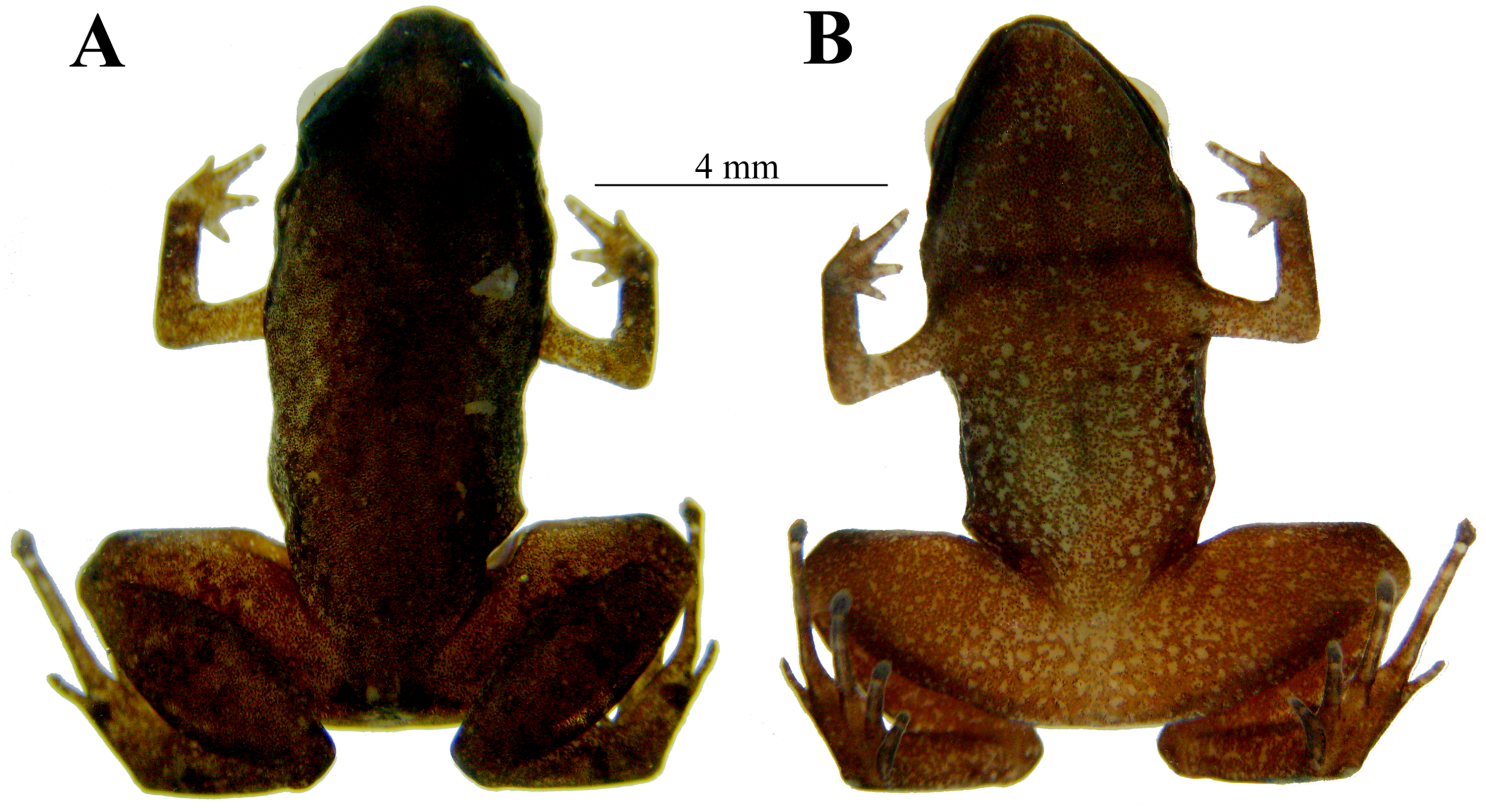
Adult holotype of *Adelophryne michelin* sp. nov. (MZUESC 17509, SVL 10.5 mm). Dorsal view (A), ventral view (B).

Adult, female (MZUESC 17509; Figs 1 and 2) collected in 8 October 2012 in the fragment Vila 5 of pristine forests (13°48’59.45” S, 39°12’13.39” W, 270 m above sea level) at Reserva Ecológica Michelin (REM), municipality of Igrapiúna by MS, CVMM and DSR.

#### Paratypes

Seven adult males collected in 8 October 2012 in the Vila 5 fragment (1 individual MBML 19497) and 15 September 2014 in the Pacangê fragment (6 individuals MBML 10495, MZUESC 17506–17508, and ZUFG 10693–10694); and eleven adult females collected in 12 August 2012 (1 individual ZUFG 10696) and 8 October 2012 in the Vila 5 fragment (2 individuals MBML 10496 and ZUFG 10697) and 15 September 2014 in the Pacangê fragment (8 individuals MBML10498–10499, MZUESC 17510–17513, and ZUFG 10695–10698). These specimens were collected by CVMM, DSR, MS and RMO in areas of pristine forests at Reserva Ecológica Michelin (REM), municipality of Igrapiúna (Vila 5 13°48’59.45” S, 39°12’13.39” W, 270 m above sea level and Pacangê 13°51’1.02” S, 39°13’54.36” W, 170 m above sea level).

#### Diagnosis

The new species is included in the subfamily Phyzelaphryninae because of the molecular evidence and by the presence of apically pointed digits; its leaf litter habitat; its terminal digits either barely or not expanded [3], and the SVL not exceeding 23 mm in SVL [5]. In addition to the results of molecular analysis, the generic assignment of *Adelophryne michelin* sp. nov. is based on the possession of a head narrower than body, cranial crests absent, small size, with subdigital pad and mucronate tip on the fingers and toes, toes III and IV with discs and mucronate tips, and terminal phalanges of toes and fingers T-shaped.

The new species can be distinguished from species in the genus *Phyzelaphryne* by the absence of subarticular tubercles on fingers, for presenting indistinct tympanum, and reduction of the phalanges in the Finger IV. *Phyzelaphryne* has subarticular tubercles, distinct tympanum and no reduction of the phalanges [1,2,4,9].

The new taxon is diagnosed by the following combination of character states: (1) snout–vent length smaller than 11.5 mm (males 7.6–9.1 mm, N = 7; females 10.0–11.4 mm, N = 12); (2) tympanum indistinct without visible membrane; (3) tympanic annulus absent; (4) dentigerous processes of vomers present; (5) fingers without terminal discs, with mucronate tips, terminal phalanges T-shaped; (6) toes with terminal discs or circumferential grooves and mucronate tips; (7) terminal phalanges of toes T-shaped and sharply reduced; (8) Finger I shorter than Finger II; (9) Finger IV with two phalanges; (10) Toe III longer than Toe V; (11) subarticular tubercles absent on the fingers and toes (subdigital pads present); (12) belly skin smooth; (13) dorsum skin smooth; (14) anal flap absent.

#### Comparison with other species

*Adelophryne michelin* sp. nov. is distinguished from all other congeners except *A*. *glandulata* and *A*. *meridionalis*, by having an indistinct tympanum (distinct in *A*. *adiastola*, *A*. *baturitensis*, *A*. *gutturosa*, *A*. *maranguapensis*, *A*. *mucronata*, *A*. *pachydactyla*, and *A*. *patamona*). It is further distinguished from *A*. *baturitensis*, *A*. *gutturosa*, *A*. *maranguapensis*, *A*. *mucronata*, and *A*. *patamona* by its smaller size (maximum SVL 16.3 mm in *A*. *baturitensis*, 16 mm in *A*. *gutturosa*, 17.4 mm in *A*. *maranguapensis*, 14.9 mm in *A*. *mucronata*, and 23 mm in *A*. *patamona*) vs. 11.4 mm in *Adelophryne michelin* sp. nov. The new species can also be distinguished from *A*. *baturitensis* and *A*. *maranguapensis* by lacking discs or circumferential grooves on fingers and from *A*. *baturitensis* by absence of subarticular tubercles in the toes (subdigital pads in *A*. *michelin* sp. nov.); from *A*. *glandulata* and *A*. *gutturosa* by the absence of a distinct glandular ridge line that runs from the posterior part of eye to the insertion of the forelimb; from *A*. *adiastola*, *A*. *glandulata*, *A*. *mucronata*, and *A*. *patamona* by smooth skin texture of dorsum (vs. shagreened to granular in *A*. *adiastola*, shagreened with small and rounded granules in *A*. *glandulata*, smooth with scattered small granules in *A*. *mucronata*, and tuberculated in *A*. *patamona*); from *A*. *meridionalis* by having toes II, III, and IV with circumferencial grooves or disc (vs. only Toe IV with circumferencial grooves in *A*. *meridionalis*); and from *A*. *mucronata* and *A*. *maranguapensis* by the absence of anal flap in *A*. *michelin* sp. nov. (anal flap present in *A*. *mucronata* and *A*. *maranguapensis*).

#### Description of the holotype

**Fig 3:**
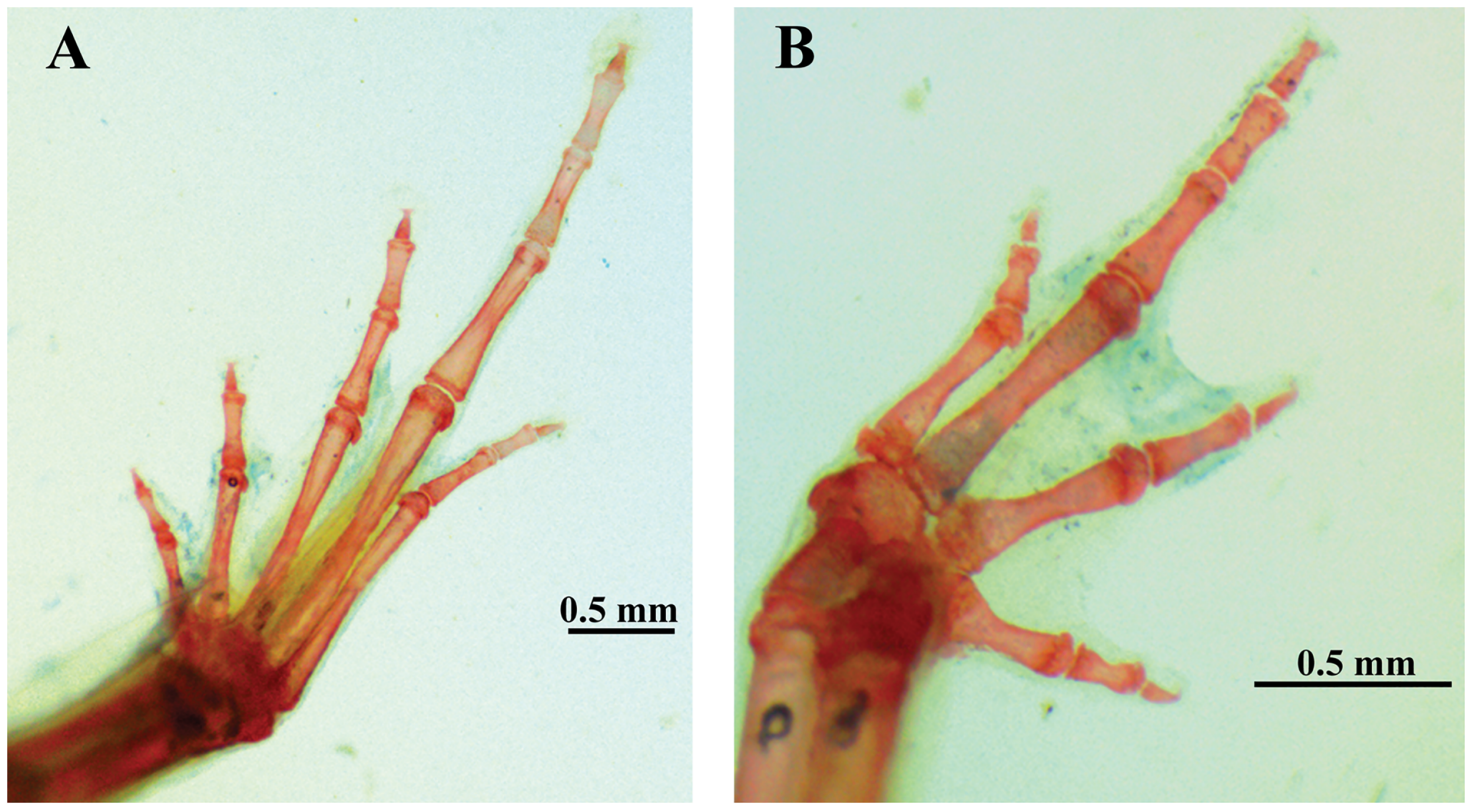
Views of foot (A) and hand (B) of a cleared and stained paratype (ZUFG 10695) of *Adelophryne michelin* sp. nov.

Adult female, SVL 10.5 mm (Figs 1 and 2). Snout rounded, slightly triangular in ventral and dorsal view (Fig 1A and 1B) and truncate, slightly rounded in lateral views (Fig 1C). ETSD larger than the ED. END smaller than the IND. Nostrils not protruding and round. IND slightly larger than the IOD. Canthus rostralis indistinct; loreal region slightly concave. Choanae small, round, located laterally. Dentigerous processes with two rows of four teeth; widely separated posterior to choanae, centraly localized. Tongue ovoid, free except its anterior margin. Pupil horizontally oval. Upper eyelid slightly convex. Temporal region vertical, tympanum indistinct. Skin texture of venter, dorsum and limbs smooth; flanks and ventral region of thighs areolate. Anal flap absent, cloacal opening horizontally positioned at slightly below the level of the dorsal surface of the thigh. Fingers without disks; fingers I, II and III with mucronate tips, Finger IV with rounded tip. Fingers thin, depressed and short, without webbing. Fingers formula: IV<I<II<III (Fig 1D). Phalangeal formula 2–2–3–2 (Fig 3B). Fingers and palm appear to be surrounded by a narrow strip of transparent skin. Subarticular tubercles absent with round subdigital pads, formula 1–2–2–1; no pads under ultimate phalanges and no supernumerary tubercles. Inner metacarpal tubercle ovoid, outer metacarpal tubercle round, slightly larger than inner. Toes without webbing, cylindrical, slightly flattened. Toes formula: I<V<II<III<IV; toes II, III and IV with discs and mucronate tips, Toe V with circumferential grooves and mucronate tips; Toe I with circumferential groove (Fig 1E). Phalangeal formula 2–2–3–4–3 (Fig 3A). Skin transparent only on the distal portion of toes II, III, and IV. Subarticular tubercles absent with round subdigital pads (0–0–2–1–0); no pads under ultimate phalanges and no supernumerary tubercles. Inner metatarsal tubercle oval; outer metatarsal tubercle smaller and rounded. For measurements see Table 1.

**Table 1:** Measurements of the type series of *Adelophryne michelin* sp. nov. Values presented in millimeters as mean ± standard deviation (range, as maximum-minimum values).

	Holotype MZUESC 17509	Paratypes	
Measurement	Female	Males (N = 7)	Females (N = 11)
SVL	10.5	8.5 ±0.5 (9.1–7.6)	10.7 ±0.4 (11.4–10.0)
HL	3.3	3.2 ±0.4 (3.6–2.5)	3.7 ±0.4 (4.1–3.1)
HW	3.8	3.1 ±0.2 (3.4–2.8)	3.7 ±0.1 (3.9–3.5)
ED	0.9	1.0 ±0.2 (1.3–0.9)	1.2 ±0.3 (1.8–0.9)
UEW	0.9	0.8±0.1 (1.0–0.6)	1.0 ±0.1 (1.2–0.8)
IOD	1.7	1.3 ±0.1 (1.5–1.1)	1.6 ±0.1 (1.8–1.4)
IND	1.2	1.0 ±0.1 (1.2–0.9)	1.2 ±0.1 (1.4–1.0)
END	1.2	0.7 ±0.1 (0.9–0.5)	1.1 ±0.3 (1.5–0.7)
NSD	0.7	0.6 ±0.1 (0.7–0.5)	0.7 ±0.1 (0.9–0.6)
ETSD	1.9	1.3 ±0.2 (1.5–1.0)	1.8 ±0.3 (2.4–1.3)
FL	7.2	6.7 ±1.1 (8.4–4.9)	7.6 ±0.5 (8.2–6.7)
THL	4.9	4.5 ±0.5 (5.1–3.8)	5.1 ±0.2 (5.5–4.8)
TL	4.8	4.1±0.5 (4.6–3.0)	4.9 ±0.2 (5.2–4.6)

#### Color of holotype in life

Coloration of holotype in life is unknown.

#### Color of holotype in preservative

Venter brown with numerous small white and black dots. Throat dark brown. Dorsum brown with numerous small white and black dots. Loreal region black, with a black stripe extending along the flanks and reaching the groin, with numerous small white dots; a black interorbital stripe in “V” shape, but the lines do not connect. Upper eyelid black. Thigh and tibia with numerous black dots that form lines (Fig 2).

#### Variation

**Fig 4:**
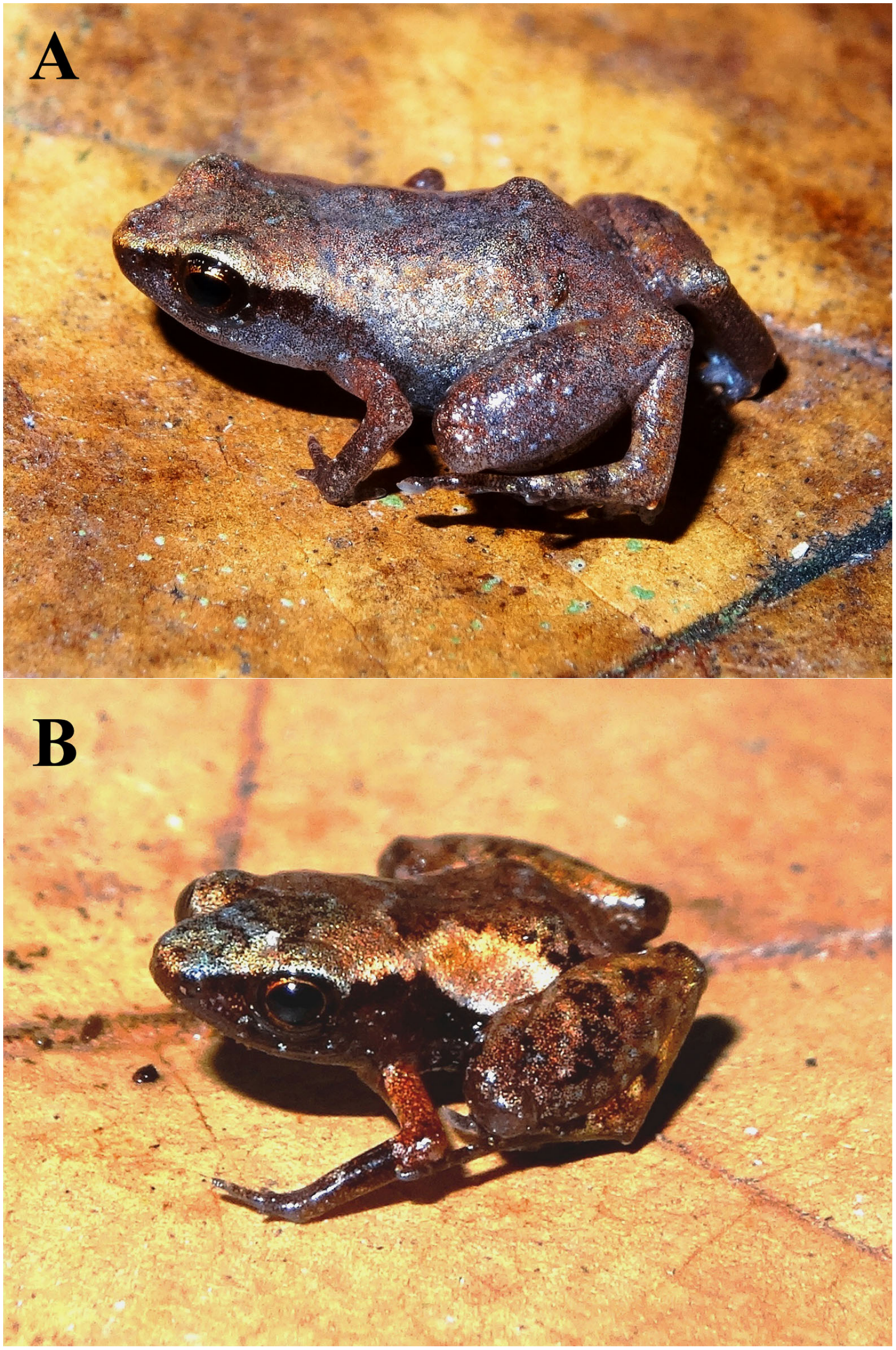
Adult individuals of *Adelophryne michelin* sp. nov. in life (A) female paratype MBML 10498 and (B) paratype but not identified. Individual (A) showing an unusual bluish coloration and (B) showing common coloration.

In life the specimens vary in dorsal coloration, though very little, showing a pattern in the dorsal coloration slightly golden (Fig 4B), but some individuals show a bluish coloration (Fig 4A) Color in life (based on non-identifiable paratypes and one female paratype [MBML 10498] of Fig 4), venter dark with numerous small white dots. Throat and underside of thighs and shanks slightly golden. Dorsum slightly golden with two dark brown stripes of various widths in the middle region in “Λ” shape. Loreal region dark brown, with a dark brown stripe extending along the flanks and reaching the groin, with numerous small white dots; a dark brown interorbital stripe, of various widths in “V” shape. Thigh and tibia with one or two dark brown band lines. Forelimbs sometimes red. Coloration of venter sometimes with scattered spots, dark or uniform. Iris reddish brown with black reticulations. Color pattern does not change in preservative, shades of colors become darker than in life. The formula of pads and discs in the fingers and toes vary in numbers. Males are smaller, and have toes IV and V are more depressed than females. For morphometric variation see Table 1. The other two species that are sympatric with *A*. *michelin* have visible tympanum.

#### Molecular relationships and divergence

**Fig 5:**
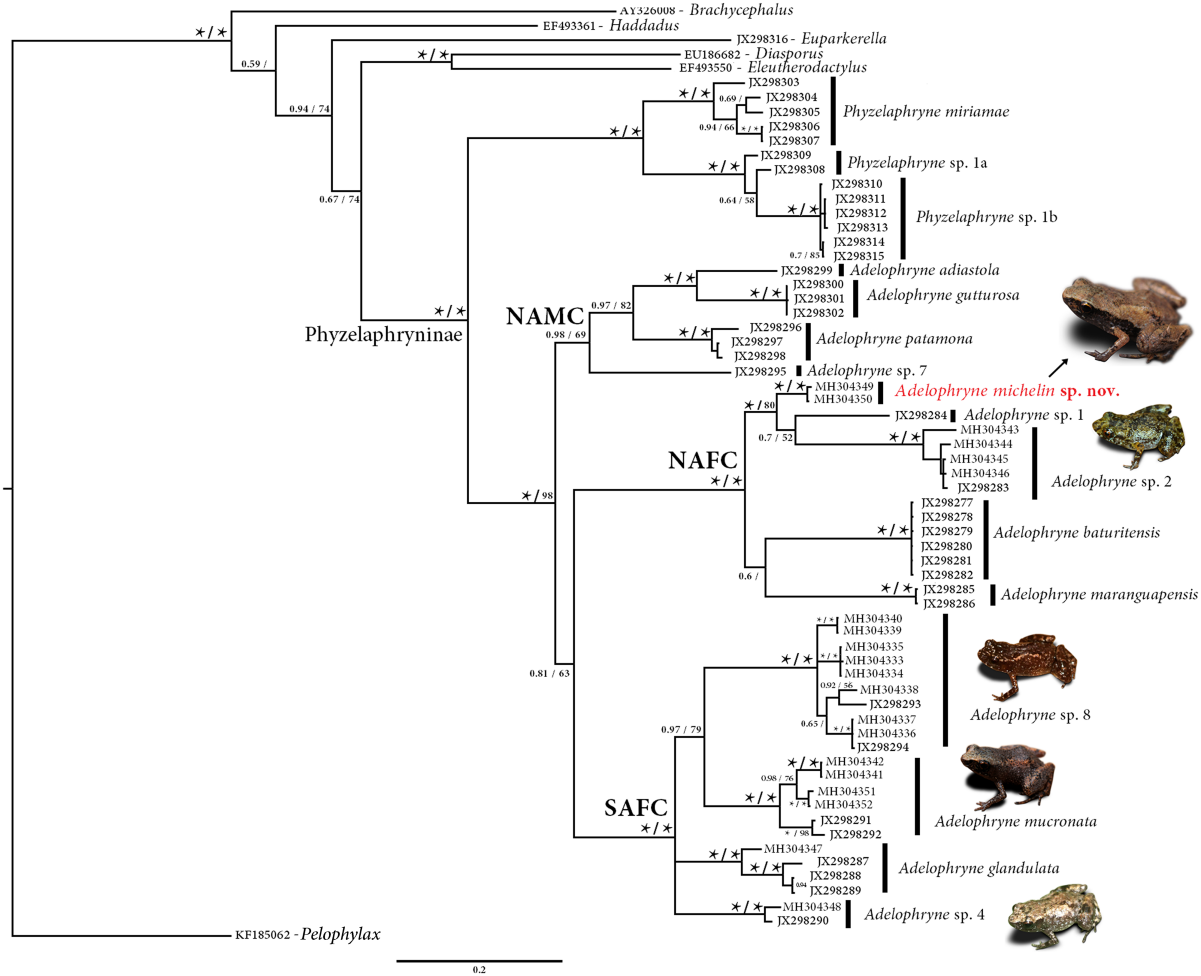
Phylogenetic relationship of genus *Adelophryne* through 16S mitochondrial rRNA fragment gene (798 bp). Bayesian Posterior Probabilities and Maximum Likehood Bootstrap values are indicated above and below the branches. Asterisk = ≥ 0.99 and values below 0.50 are not shown (see methods for analysis details). Abbreviations are: NAFC = Northern Atlantic Forest Clade; NAMC = Northern Amazonia Clade and SAFC = Southern Atlantic Forest Clade representing the clades proposed by Fouquet et al. [9]. The paratype of *Adelophryne glandulata* (MZUESC 12180) has number MH304347 in the tree. Photos not to scale.

The Maximum Likelihood and Bayesian methods resulted in similar phylogenetic trees (Fig 5). *Adelophryne michelin* sp. nov. is allocated in the North Atlantic Forest Clade (NAFC) as defined by Fouquet et al. [9] and is sister to a clade formed by *Adelophryne* sp. 1 and *Adelophryne* sp. 2; this clade in turn is sister to a clade with the other species of the NAFC (*A*. *baturitensis* and *A*. *maranguapensis*). Divergences (uncorrected pairwise distances) for the mitochondrial 16S rRNA gene of *A*. *michelin* sp. nov. to other species of the genus range between 10.0 to 22.9%, being lowest to *Adelophryne* sp. 1 from Caruaru, Pernambuco (Table 2). Based on the phylogenetic position and high molecular divergence, we conclude that *Adelophryne michelin* sp. nov. does not represent any of the candidate species listed by Fouquet et al. [9].

**Table 2:** Uncorrected p–distances in percent, calculated for DNA sequences of a fragment of the mitochondrial 16S rRNA gene (365 bp) among species of *Adelophryne*.

	**Species**	**Intraspecific** **p-distance**	**1**	**2**	**3**	**4**	**5**	**6**	**7**	**8**	**9**	**10**	**11**	**12**	**13**
**1**	*A*. *adiastola* (JX298299)	-	-												
**2**	*A*. *baturitensis* (JX298279)	0.0	21.1	-											
**3**	*A*. *glandulata* (MH304347)	3.6	19.5	22.5	-										
**4**	*A*. *gutturosa* (JX298302)	0.0	10.8	21.1	20.1	-									
**5**	*A*. *maranguapensis* (JX298285)	0.0	23.4	16.8	21.6	23.2	-								
**6**	*A*. *michelin* sp. nov. (MH304349–50)	0.2	19.9	13.9	21.2	20.9	14.5	-							
**7**	*A*. *mucronata* (JX298291)	3.4	19.2	22.3	12.6	19.8	20.7	20.3	-						
**8**	*A*. *patamona* (JX298296)	1.4	14.0	20.1	18.6	12.8	20.2	19.0	16.8	-					
**9**	*A*. sp. 1 (JX298284)	-	21.3	17.3	19.9	22.0	16.6	10.0	20.2	21.9	-				
**10**	*A*. sp. 2 (JX298283)	1.7	23.0	18.5	25.2	21.3	19.9	14.6	25.1	22.0	16.5	-			
**11**	*A*. sp. 4 (JX298290)	1.5	19.2	21.4	12.3	18.7	22.4	18.9	13.1	16.8	19.9	21.8	-		
**12**	*A*. sp. 7 (JX298295)	-	19.0	23.1	17.7	19.2	21.4	22.0	17.7	15.1	23.1	26.2	14.5	-	
**13**	*A*. sp. 8 (JX298294)	2.4	20.4	23.8	14.6	19.0	21.9	22.9	14.6	16.9	23.4	25.5	14.0	18.4	-

**Fig 6:**
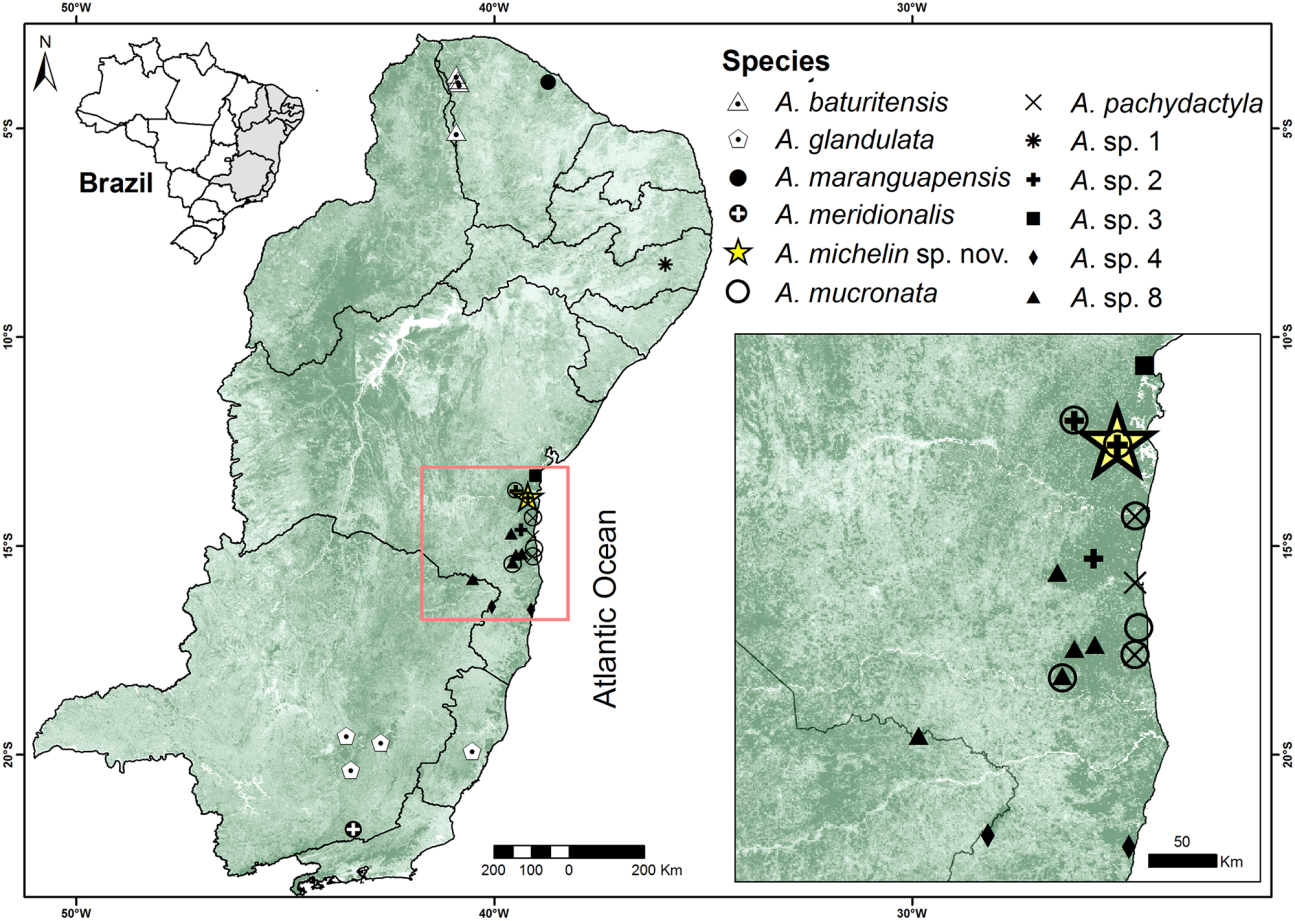
Geographic distribution of *Adelophryne* species in eastern Brazil. Yellow star = type locality of *Adelophryne michelin* sp. nov. in municipality of Igrapiúna (Reserva Ecológica Michelin), Bahia State, Brazil.

Our results recover a paratype of *Adelophryne glandulata* (tree number MH304347; MZUESC 12180) with *A*. sp 5 (JX298287, JX298288, JX298289 *sensu* Fouquet et al. [9]). In addition, we report new localities (Fig 6) and information on the clades defined by Fouquet et al. [9]. The samples from Ilhéus, Wenceslau Guimarães and Igrapiúna were placed with *Adelophryne* sp. 2; samples from Igrapiúna and Wenceslau Guimarães were placed with *A*. *mucronata* (*Adelophryne* sp. 6 *sensu* Fouquet et al. [9]); specimens collected in Almadina (Serra do Corcovado), Camacan (Serra Bonita), Arataca (Serra das Lontras) and Macarani (RPPN Mata do Passarinho) were placed with *A*. *pachydactyla*; and one specimen of Guaratinga (Parque Nacional do Alto do Cariri) was clustered in the *Adelophryne* sp. 4 clade.

#### Geographic distribution

*Adelophryne michelin* sp. nov. is known only from the type locality, at the Reserva Ecológica Michelin (REM), municipality of Igrapiúna, Bahia—Brazil (Fig 6).

#### Natural history, ecology and status conservation

*Adelophryne michelin* sp. nov. occurs in the leaf litter of primary forest. Two large ovarian eggs (2.0 mm) were found in one female of *Adelophryne michelin* sp. nov. (ZUFG 10697). We dissected five specimens of *Adelophryne michelin* sp. nov. one specimens there was nothing (ZUFG 10696) and four specimens revealed ants in their stomachs (ZUFG 10695 and 10697, MZUESC 17506, MBML10498). Beetles were found in stomachs of *A*. *glandulata* and ants were also found in *A*. *glandulata* [8] in *A*. *gutturosa* [5] and in *A*. *mucronata* [6]. We recorded a new population of *A*. *mucronata* and *A*. sp. 2 (*sensu* Fouquet et al. [9]), both species living sympatrically and syntopically with *A*. *michelin* sp. nov. in the REM.

*Adelophryne michelin* sp. nov has only been recorded at the type locality, in the Atlantic Forest biome of southeast Bahia, being restricted to well preserved forests. Based on the forest remnants size of landscape its area of occupancy is <500 km². As such, this new species can be included under criterion B2a of IUCN Red List [28]. Because we do not have data on habitat decline [11] or population data, we felt unable to fit the species into a threat category given that at least two of three conditions of criterion "B" need to be fulfilled for including a species into a threat category. Thus, we suggest that *Adelophryne michelin* sp. nov. should be listed as Near Threatened (NT) under the criterion B2a.

#### Geographic distribution remark

We report another new locality for *A*. *mucronata* in Estação Ecológica Wenceslau Guimarães in the municipality of Wenceslau Guimarães, increasing the known geographic distribution of this species by approximately 110 km north from its nearest locality (straight line), municipality of Itacaré, Bahia State [6]. We furthermore extend the geographic distribution of *A*. *glandulata* approximately 210 km west from its type locality, Santa Tereza, Espírito Santo State [8], to Mariana, Catas Altas, and Marliéria municipalities, and Serra do Cipó, Minas Gerais State (Fig 6).

#### Phylogenetic remark

Santana et al. [7] suggested that *Adelophryne* sp 5 (*sensu* Fouquet et al. [9]) could represent *A*. *meridionalis*. However, the morphological examination of individuals of the same population of *A*. sp. 5 (Mariana, Catas Altas, and Marliéria municipalities, Serra do Cipó, Minas Gerais State see S1 Appendix) used by Fouquet et al. [9], allowed to define *A*. sp. 5 as *A*. *glandulata*, as well as our molecular results where the sample of the paratype of *A*. *glandulata* analyzed (MZUESC 12180—tree number MH304347) was placed in the same clade. Also, Fouquet et al. [9] tentatively assigned the populations of Serra do Teimoso (Jussari) and Serra das Lontras (Arataca) to the nominal species *A*. *pachydactyla* due to the geographical proximity to the type locality and the morphological characteristics compatible with this species. The authors did not detail the morphological evidence that led them to reach this conclusion. *Adelophryne pachydactyla* was described based on a single diminutive specimen with SVL of 11.1 mm and a presence of two phalanges in the finger IV, as a more remarkable morphological character [4]. An individual collected in Serra Bonita (tree number MH304334), recovered in the same clade assigned as *A*. *pachydactyla* from Fouquet et al. [9] (tree number JX298293 and JX298294). However, the individual was cleared and stained and has three phalanges on the IV finger (see Figure A in S3 Appendix), characteristic that differs from to *A*. *pachydactyla*. Therefore, we consider that the clade assigned as *A*. *pachydactyla* from Fouquet et al. [9] represents another candidate species, which we treat here as *Adelophryne* sp. 8 (under description by Lourenço-de-Moraes et al. in prep.), one of the largest species of the genus (SVL between 13–16 mm), and widely distributed in the mountain forests of southern Bahia. Therefore, the current phylogenetic position of *A*. *meridionalis* and *A*. *pachydactyla* remains uncertain.

#### Nomenclatural remark

The generic name *Adelophryne* is feminine in gender [1] and the species name *A*. *mucronatus* thus needs to be corrected to *A*. *mucronata*, as already used in the previous sections of the present study.

*Adelophryne mucronata* Lourenço-de-Moraes, Solé & Toledo, [6].

*Adelophryne mucronatus* Lourenço-de-Moraes, Solé & Toledo, [6]: 61.

## Discussion

The genus *Adelophryne* contains some of the smallest anurans in the world and its diversity is vastly underestimated. Although Fouquet et al. [9] revealed a high number of candidate species of *Adelophryne*, the same authors highlighted that these results represented a tiny portion of the real potential distribution of the genus, especially due to the low number of sampled localities. Many of these candidate species were known only from one or two localities and in our study we report new populations for almost all of them.

Miniaturization in anurans is accompanied by reductions in the number of digits and phalangeal elements, and the loss or reduction of some cranial elements [29,30]. Some studies have shown high patterns of diversity in miniaturized species [31,32,33]. For example, in the genus *Brachycephalus*, the tolerance to certain environmental conditions seems to play an important role in the high diversity and levels of microendemism [34,35]. Miniaturization might have exerted important consequences on the diversification of *Adelophryne* given that the diminutive species have a high surface to volume ratio and are thus more susceptible to desiccation [36]. This might have led to restricted gene flow among populations and thus accelerated genetic differentiation as hypothesized for other frogs [37], and might have restricted these species to humid forest where such genetic differentiation is more commonly observed [38]. We hypothesize these factors might ultimately have contributed to the large number of species with small ranges seen today in *Adelophryne*.

Species of the genus *Adelophryne* are sensitive to anthropogenic changes and live exclusively in forest areas [6,8,15]. Conforming to this pattern, also *A*. *michelin* sp. nov. was collected in a private protected area in pristine forest. The species is characterized by a small distribution range and might be sensitive to habitat degradation. Their diminutive size may limit their dispersion in fragmented landscapes which may compromise the genetic variability of populations to long-term. Lourenço-de-Moraes et al. [8] and Ferreira et al. [39] have shown that *Adelophryne* species may be sensitive to edge effect. The conservation area in REM has a relatively small pristine forest area (<1800 ha see above in MM), being of great importance for the conservation of this diminutive species. Fieldworks were carried out in the surround landscape, and we do not have been successful in the record of the species (IRD and CVMM pers. obs.). We encourage field trips to find new populations, as well as studies to understand their population trends and whether there is any interruption of gene flow among the subpopulations caused by habitat fragmentation. This information is crucial for a better definition of its conservation status in accordance with IUCN criteria.

*Adelophryne michelin* sp. nov. was recovered as member of the North Atlantic Forest Clade (NAFC) of *Adelophryne*. The other species in this clade, *A*. *baturitensis*, *A*. *maranguapensis* and *Adelophryne* sp. 1 (identified as *A*. *baturitensis*; see Loebman et al. [40]) agree with each other (and differ from the new species) in various morphological features such as SVL greater than 12 mm, three phalanges in finger IV, distinct tympanum, subarticular tubercles and distinct enlarged terminal discs on fingers. Two other NAFC candidates (*Adelophryne* sp. 2 and sp. 3) have not yet been morphologically studied and their possible morphological differentiation remains unresolved. Despite its strongly supported phylogenetic position as member of the NAFC the new species has morphological features more similar to the species of the South Atlantic Forest Clade (SAFC) as *A*. *glandulata*. Considering this new evidence, now both the clades NAMC and NAFC include representatives with similar patterns of phalangeal reduction (*A*. *adiastola* and *A*. *michelin* sp. nov.) as the species in SAFC, suggesting extensive homoplasy.

Herein, we described a new miniature species of *Adelophryne*, highlighting that the diversity within the genus is still underestimated. Furthermore, we report for the first time the occurrence of at least three species of the genus under syntopic or sympatric conditions; new localities for all candidate species (exception for *Adelophryne* sp. 3) listed by Fouquet et al. [9]; and unravel a new candidate species (*Adelophryne* sp. 8) from the Atlantic Forest. Thus, we encourage the collection of more field data and the use of an integrative taxonomic approach based on both morphological and molecular data for supporting the description of the candidate species [9], as a full species inventory is crucial for a better understanding of both the diversity and the evolution of morphological characters within the genus.

## Supporting information

S1 Appendix Specimens examined. (DOCX)

S2 Appendix Numbers of GenBank accession for comparative 16s mitochondrial rRNA fragment sequences used in phylogenetic analysis (as in Fouquet et al. [9]). (DOCX)

S3 Appendix Figure A. Cleared and stained individual of *Adelophryne* sp 8 (*Adelophryne pachydactyla—sensu* Fouquet et al [9]). (DOCX)
