# Supplementary material for: Diversity of miniaturized frogs of the genus Adelophryne (Anura: Eleutherodactylidae): A new species from the Atlantic Forest of northeast Brazil
Source: PLoS One. 2018 Sep 19;13(9):e0201781. doi: 10.1371/journal.pone.0201781 (PMC6145526; doi:10.1371/journal.pone.0201781)
Supplement: S1 Appendix — (DOCX) [file pone.0201781.s001.docx]

**S1 Appendix**

**Specimens examined:**

*Adelophryne adiastola* – Ecuador: **Pastaza:** Kurintza (División de Herpetología, Museo Ecuatoriano de Ciencias Naturales, Quito, Ecuador) DHMECN 4378;

*Adelophryne baturitensis* – Brazil: **Ceará:** Guaramiranga CFBH 20469–76; Tiangua CFBH 24554–67; Viçosa do Ceará CFBH 24579–85, NUROF-UFC 3912, 3696;

*Adelophryne glandulata* – Brazil: **Espírito Santo**: Santa Teresa (Reserva Biológica Santa Lúcia) MNRJ 28344–34932, (Reserva Biológica Augusto Ruschii) MBML 7636–7, MNRJ 87081, MZUESC 12178 and 12180, ZUFG 7961–2. **Minas Gerais:** Mariana (Mata estrada Mariana - Catas Altas) UFMG 4108, 4115, 4117–8, (Mina de Fabrica Nova Vale S.A) UFMG 11643–4, 11646, 11751, 11755; Catas Altas (ADA PDE Trevo. Margem estrada Catas Altas-Mariana) UFMG 8110, 8127, 8140, 8157–60; 8167–68;

*Adelophryne maranguapensis* – Brazil: **Ceará**: Maranguape: CFBH 24515–27, NUROF-UFC 3810, 3745;

*Adelophryne meridionalis* – Brazil: **Minas Gerais**: Juiz de Fora (Parque Municipal de Lajinha) MZUFV 12625;

*Adelophryne mucronata* – Brazil: **Bahia**: Una CFBH 23672, ZUEC 6169, 12139, 16626, MZUESC 9091–96; Igrapiúna MZUESC 19140–41; Wenceslau Guimarães MZUESC 19149–50;

*Adelophryne pachydactyla* – Brazil: **Bahia**: Itacaré (RPPN Capitão) ZUEC 18212–13 and Una (RPPN Nova Angélica) ZUEC 17825.
