# Supplementary material for: Diversity of miniaturized frogs of the genus Adelophryne (Anura: Eleutherodactylidae): A new species from the Atlantic Forest of northeast Brazil
Source: PLoS One. 2018 Sep 19;13(9):e0201781. doi: 10.1371/journal.pone.0201781 (PMC6145526; doi:10.1371/journal.pone.0201781)
Supplement: S2 Appendix — (DOCX) [file pone.0201781.s002.docx]

**S2 Appendix**

**Numbers of GenBank accession for comparative 16s mitochondrial rRNA fragment sequences used in phylogenetic analysis (as in Fouquet et al. [9]).**

*Adelophryne* sp. 1: Brazil: **Pernambuco**: JX298284.

*Adelophryne* sp. 2: Brazil: **Bahia**: JX298283, MH304343–MH304346.

*Adelophryne* sp. 4: Brazil: **Bahia**: JX298290, MH304348.

*Adelophryne* sp. 7: Brazil: **Amapá**: JX298295.

*Adelophryne* sp. 8: Brazil: **Bahia**: JX298294, JX298293, MH304333–MH304340.

*Adelophryne adiastola*: Colombia: JX298299.

*Adelophryne baturitensis*: Brazil: **Ceará:** JX298277, JX298278, JX298279, JX298282, JX298280, JX298281.

*Adelophryne* *glandulata*: Brazil: **Minas Gerais**: JX298288, JX298289, JX298287; **Espirito Santo**: MH304347.

*Adelophryne gutturosa*: Guyana: JX298302, JX298301; Venezuela: JX298300.

*Adelophryne maranguapensis*: Brazil: **Ceará**: JX298285, JX298286.

*Adelophryne michelin* **sp. nov**: Brazil: **Bahia**: MH304349-MH304350.

*Adelophryne* *mucronata*: Brazil: **Bahia**: JX298291, JX298292, MH304341–MH304342, MH304351–MH304352.

*Adelophryne patamona*: Guyana: JX298296, JX298298, JX298297.

*Phyzelaphryne* sp. 1a: Brazil: **Amazonas**: JX298309, JX298308.

*Phyzelaphryne* sp. 1b: Colombia: JX298310, JX298314, JX298313, JX298315, JX298311, JX298312.

*Phyzelaphryne miriamae*: Brazil: **Amazonas:** JX298303, JX298307, JX298306, JX298304, JX298305, EU186689.

*Brachycephalus ephippium*: Brazil: AY326008.

*Diasporus diastema*: Costa Rica: EU186682.

*Eleutherodactylus coqui*: Puerto Rico: EF493550.

*Euparkerella brasiliensis*: Brazil: **Rio de Janeiro**: JX298316.

*Haddadus binotatus*: Brazil: EF493361.

*Pelophylax nigromaculatus*: China: KF185062.
