# Supplementary material for: Diversity of miniaturized frogs of the genus Adelophryne (Anura: Eleutherodactylidae): A new species from the Atlantic Forest of northeast Brazil
Source: PLoS One. 2018 Sep 19;13(9):e0201781. doi: 10.1371/journal.pone.0201781 (PMC6145526; doi:10.1371/journal.pone.0201781)
Supplement: S3 Appendix — (DOCX) [file pone.0201781.s003.docx]

**S3 Appendix**

*Adelophryne* sp8.

*Adelophryne pachydactyla* (*sensu* Fouquet et al. [9])


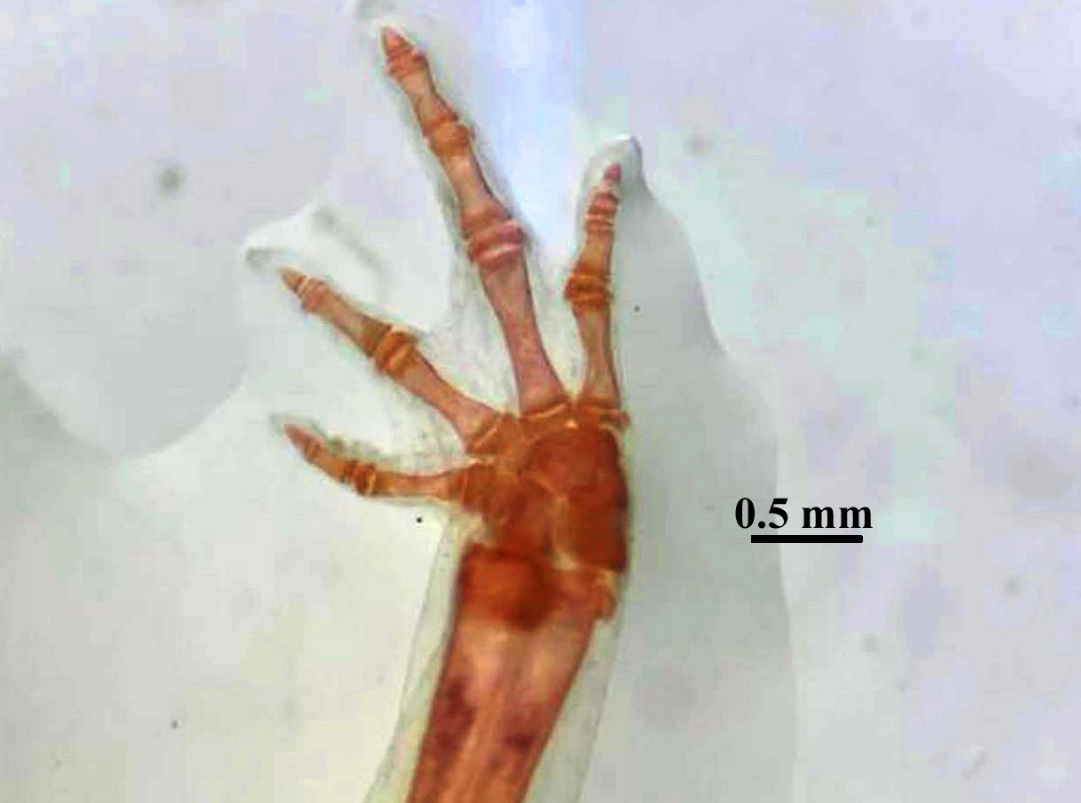


**Figure A.** Views of hand of a cleared and stained (MZUESC 19143) of *Adelophryne* sp. 8.
